# Essential medicines in Tanzania: does the new delivery system improve supply and accountability?

**DOI:** 10.1057/hs.2013.14

**Published:** 2013-11-15

**Authors:** Inez Mikkelsen-Lopez, Peter Cowley, Harun Kasale, Conrad Mbuya, Graham Reid, Don de Savigny

**Affiliations:** 1 Swiss Tropical and Public Health Institute, Basel, Switzerland; 2 University of Basel, Basel, Switzerland; 3 Abt Associates, Bethesda, U.S.A.; 4 PRAXIS Consultants, Dar es Salaam, Tanzania

**Keywords:** medicines supply, supply chain management, health systems, information system, accountability Tanzania

## Abstract

*Objective*: Assess whether reform in the Tanzanian medicines delivery system from a central ‘push' kit system to a decentralized ‘pull' Integrated Logistics System (ILS) has improved medicines accountability. *Methods*: Rufiji District in Tanzania was used as a case study. Data on medicines ordered and patients seen were compiled from routine information at six public health facilities in 1999 under the kit system and in 2009 under the ILS. Three medicines were included for comparison: an antimalarial, anthelmintic and oral rehydration salts (ORS). *Results*: The quality of the 2009 data was hampered by incorrect quantification calculations for orders, especially for antimalarials. Between the periods 1999 and 2009, the percent of unaccounted antimalarials fell from 60 to 18%, while the percent of unaccounted anthelmintic medicines went from 82 to 71%. Accounting for ORS, on the other hand, did not improve as the unaccounted amounts increased from 64 to 81% during the same period. *Conclusions*: The ILS has not adequately addressed accountability concerns seen under the kit system due to a combination of governance and system-design challenges. These quantification weaknesses are likely to have contributed to the frequent periods of antimalarial stock-out experienced in Tanzania since 2009. We propose regular reconciliation between the health information system and the medicines delivery system, thereby improving visibility and guiding interventions to increase the availability of essential medicines.

Key Messages • The transition from a ‘push' to a ‘pull' medicines delivery system in Tanzania was designed in part to increase accountability. At a district level, we found no evidence of an overall improvement in this dimension of medicines supply. • Weaknesses in the design and governance of the ‘pull' system include the lack of reconciliation between the medicines delivery system and the health information system, as well as requiring complex calculations to be manually completed by health workers every 3 months in a paper-based system.

## Introduction

The effective delivery of medicines requires integration and coordination of the entire health system. Policies are needed that shape the supply systems and its processes; financial systems are needed to purchase medicines; trained human resources are needed for procurement and delivery; health information systems (HISs) are needed to identify which diseases are prevalent (and therefore the extent of need); and finally governance is necessary at all levels to provide oversight and ensure overall availability and accountability of resources in the system. Medicines play an integral role in the performance of the health system (Ruxin *et al*, 2005; Roberts & Reich, 2011); therefore, losses of essential medicines are not only a public health issue, but are an overall indicator of the ability of the health system to deliver adequate and quality health care. Deficits of medicines represent a direct loss of resources, particularly concerning in low-income countries like Tanzania where medicines along with medical supplies constitute the largest discretionary spending in health and account for approximately 10% of total health expenditure (Euro Health Group, 2009).

The aim of this study was to investigate how the medicines delivery system has changed in Tanzania over the past decade moving from a ‘push' to a ‘pull' system. Our focus was to track the accountability of both systems by following tracer medicines to establish whether the governance of the medicines delivery system has been strengthened as a result of this transition.

## Background

### Essential drug program (EDP) kit (‘push' system) 1983–2008

Despite the various waves of decentralization experienced in Tanzania during the early 1990s, medicines and other supplies were still centrally provided (‘push') as standard, pre-packed EDP ‘kits' to all health facilities (excluding hospitals) from the Medical Stores Department (MSD). The MSD is the national semi-autonomous, non-profit department under the Ministry of Health and Social Welfare (MoHSW), responsible for the procurement and delivery of medicines to public and Non-Governmental Organization health facilities. Kits and the MSD were established with help from the Danish International Development Agency, DANIDA, together with UNICEF and the Government of Tanzania. Kits were colored either blue or yellow depending on the level of health facility (dispensary or health center, respectively) and delivered six times a year (two kits per delivery) (Amenyah *et al*, 2005). Each kit was designed to last a month, and as they were procured pre-packed from both international and national suppliers (Euro Health Group, 2007a) the MSD only had to manage up to four variants (Center for Pharmaceutical Management, 2003). The kits contained 35 medicines, 17 medical supply items and 5 stationary items (United Republic of Tanzania, 1998). Medicines were selected based on a combination of the National Essential Drug List of Tanzania (first created in 1991 and updated in 2006) together with national morbidity data. The MSD delivered kits to the district capital, which had 2 weeks to distribute the kits to health facilities ensuring their arrival on the first day of the month (Euro Health Group, 2007a). A study in 1998 found that nearly all (99%) of the kits distributed arrived at their destination, suggesting few were being lost during delivery (Price Waterhouse Coopers Tanzania, 1999). Nevertheless, the standardized nature of the kits meant that in some areas certain medicines were depleted at a faster rate, causing stock-outs or accumulated surpluses due to differences in catchment areas and disease burdens (Center for Pharmaceutical Management, 2003; Amenyah *et al*, 2005; COWI *et al*, 2007). To mitigate stock-outs and expired medicines, the District Medical Officer (DMO) was authorized to reallocate medicines between facilities; however, because of lack of funds for transport and significant political pressure by communities not to move medicines away from their local facilities, redistribution of medicines seldom occurred (Gilson *et al*, 1994).

### Indent/integrated logistics system (ILS) (‘pull' system) 2004–present

In early 2000, with support from DANIDA, the Pharmaceutical Supply Section (PSS) within the MoHSW designed a new ‘pull' system (indent), which included 70 essential medicines and allowed health facilities (excluding hospitals) to specifically order individual medicines. Vertical programs such as family planning and specific disease control programs including sexually transmitted infections, malaria and HIV remained independent and developed their own individual supply chains. Under the indent system, facilities had individual accounts at MSD and received a standard credit roughly equivalent to three monthly kits worth every quarter (Boex & Msemo, 2007). Health facilities were required to estimate quarterly consumption (current ‘stock on hand' subtracted from quarterly monthly consumption) for the 70 items and place quarterly medicines orders through the district office. The DMO was responsible for examining the orders against the available fund credit and then distributing the packages upon receipt from MSD. The indent system meant that MSD moved away from supplying four stock items to individually packing 70 products in the orders for over 3000 health facilities every month (Center for Pharmaceutical Management, 2003). As with the kit system, health facilities were almost entirely dependent on the MSD for medicine supplies; a study carried out in 2005/2006 found little difference in medicines availability between the two systems (Euro Health Group, 2007a).

**Figure 1:**
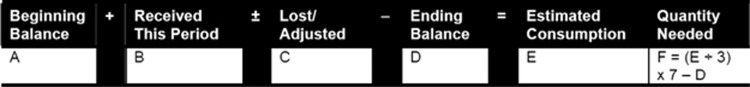
Ordering formula used in ILS R&R forms, Tanzania. *Source*: Amenyah *et al*, 2005

In 2005, the MoHSW in collaboration with John Snow Inc's DELIVER Project expanded the indent ‘pull' system to include all vertical programs under the umbrella of the ILS and rolled it out nationally in 2009. The Expanded Program of Immunization and the National Tuberculosis and Leprosy Programs were excluded, however, as they were deemed to perform well under their own vertical programs (Amenyah *et al*, 2005). The ILS introduced a new ordering system of 12 forms to be completed by health facilities. The Request and Report (R&R) form (Figure 1) is used for quarterly ordering of around 100 pre-determined priority medicines (all items in the kit were included in this list). The R&R form contains a fixed algorithm that requires data from stock ledgers together with physical counts of inventory to estimate *presumed* quarterly consumption that is subsequently used to estimate the quantity needed.

From the R&R form, the quantity needed (*F*) is estimated using the quarterly consumption (*E*) divided by three to attain the monthly consumption, which is multiplied by seven and from which any stock on hand (*D*) is deducted. Health facilities order for 7 months in advance: for the 3 months in a quarter, 2 months for the MSD and district processing time and the remaining months as a buffer stock to account for increases in consumption due to seasonal patterns and any delays in ordering (Amenyah *et al*, 2005). The quantity requested is based on quantity needed (*F*) and the MSD sale catalogue, which contains information on pack sizes. The ILS therefore increased both the number of medicines ordered and the complexity of the ordering formula.

Completed R&R forms are submitted to the district for review by both the District Pharmacist and the DMO before being sent to the MSD. Copies of the R&R form are kept at the health facility, the office of the DMO and the MSD. At the district level, health facilities are divided into three ordering groups submitting R&R forms for the quarter in different months at different periods to ease the packaging and processing load at the MSD.

Funds for the purchase of medicines represent a combination of the district block grant (from government) and a ‘basket fund' (from donors). The PSS is responsible for providing oversight on medicines policy and assisting health facilities to order medicines using the ILS. Allocation of funds for medicines purchase is based on service population. On the basis of the recommendations of PSS, funds are released quarterly by the Ministry of Finance and Economic Affairs (MoFEA) to health facility accounts in the MSD via the MoHSW. Funding for medicines is based on a revolving fund, where once health facilities place orders with the MSD the funds are released from the individual accounts and transferred to the MSD working capital, which can be used for future procurement. The MSD finances its operating costs by charging a 17.4% mark-up on all medicines and supplies, except for vertical programs where the overhead is lower at 14% (Euro Health Group, 2007b). Health facilities therefore rely almost entirely on delivery from the MSD, which in turn is reliant on the timely and complete allocation of funds from the MoHSW, which relies on the release of funds from the MoFEA.

### MTUHA/Health management and information system

At the national level, the forecasts of the demand for selected medicines is based on the data collected by the HIS (or MTUHA as known in Tanzania). The current MTUHA developed in the early 1990s (Ministry of Health and Social Welfare Tanzania, 2010) requires health facilities to manually record data in 12 booklets, which contain forms and registers. This information is summarized quarterly and submitted to the office of the DMO for review before being computerized and made accessible at the regional level. As the MTUHA system was deemed inadequate for some large programs, a number of parallel, vertical information systems for specific diseases such as HIV/AIDS, tuberculosis and leprosy have subsequently been developed (Ministry of Health and Social Welfare Tanzania, 2007).

Tanzania therefore has two sources of information for monitoring medicines accountability; on the demand side they have the HIS (MTUHA), while data on the supply of medicines come from the ILS at present, and previously from the EDP kits.

We reconcile these two sources of information from both medicines delivery systems to determine whether the accountability of medicines delivery has improved under the ILS.

## Methods

### ‘Push' – 1999

Our case study is based in the Rufiji District in South East Tanzania, one of the 132 districts of Tanzania. The Rufiji District is representative of a rural coastal district in Tanzania and the district selected by the MoHSW for the coastal sentinel demographic surveillance system. In 1999, the Rufiji District, as in the rest of the country, was receiving medicines through the ‘push' kit system. At the same time, Rufiji was one of the two pilot districts (along with Morogoro District) selected for the ‘Tanzania Essential Health Interventions Project' (de Savigny *et al*, 2008), which sought to apply the principles and methods of the *1993 World Development Report* on evidence-based planning to guide strategic investments in health based on burden of disease and cost-effectiveness analyses. We went to health facilities to collect individual patient data from ledger books on patient attendances, diagnoses and corresponding medicines dispensed at the health facility level. The data collected from facility ledger books were also compared with summary statistics compiled at the facility under the MTUHA. Data on medicines stock levels were taken from health facilities as the sum of the opening balance inventory carried over to 1999 from the previous year plus the total amount of medicines received in the kits (including any other additional medicines received) minus stock on hand at the end of the year compensating for any expired medicines removed from inventory during the year. From this, the amount of medicines specifically dispensed was compared to identify the fraction of unaccounted medicines (any consumed medicines that could not be accounted for inpatient registers). For 1999, this was conducted in 6 of the 53 public health facilities in Rufiji using a total of 11 ‘tracer' medicines: mebendazole; metronidazole; ferrous sulfate; penicillin V; magnesium trisilicate; chloroquine; doxycyline; tetracyclin ointment; aminophylline; and oral rehydration salts (ORS).

### ‘Pull' – 2009

In 2009, we replicated the analysis for unaccounted medicines in the same six health facilities to determine whether the amount of unaccounted medicines had changed following Rufiji's move, like the rest of the country, to ILS with training completed in 2009. We used data available at the district level from both the ILS and the MTUHA. As a proxy for medicines dispensed, we used the estimated consumption recorded in the ILS orders. We verified the reported estimated consumption figure by re-doing the arithmetic using the data provided (as part of Figure 1). As we were unable to obtain information about medicines dispensed, we restricted the sample medicines to those that were uniquely prescribed for a single disease, therefore assuming that the medicines would only be used for treatment of a single disease: artemisinin combination therapies (ACT) as the first-line treatment for malaria; albendazole (current anthelmintic) for the treatment of all protozoan infections; and ORS for diarrhea.

From MTUHA, we obtained annual summaries of outpatient data collected at the health facility for malaria, worms and diarrhea.

## Results

### Reconciliation of medicines supply under the ‘push' system, 1999

**Figure 2:**
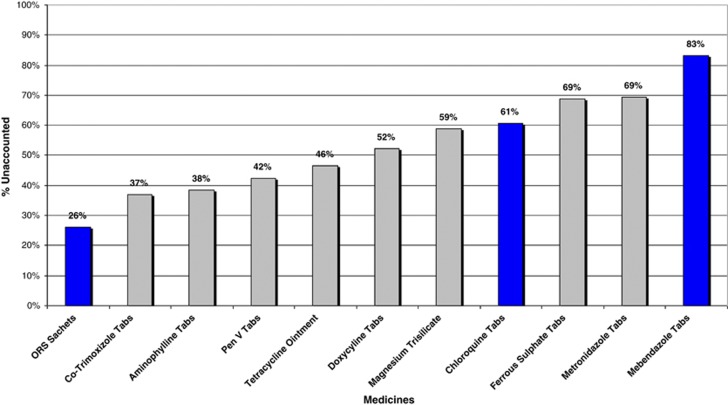
Reconciliation of medicines received *vs* medicines dispensed, Rufiji, 1999. Sample of two health centers and five dispensaries. Dark bars represent medicines for tracer disease followed in 2009.

The 1999 results illustrate that the summary health information reported under the MTUHA was accurate, with less than 1% difference in total outpatient numbers compared with the information collected from the patient ledgers. Figure 2 illustrates that there were important disparities between recorded amounts of medicines dispensed and outpatients recorded for all 11 medicines investigated. We could not account for over 50% of medicines received in 1999; this was most evident in the case of mebendazole where almost the entire stock (83%) was unaccounted for.

### Reconciliation of medicines supply under the ‘pull' system, 2009

Data gaps existed in the completion of R&R forms where not one of the six health facilities submitted all four forms in 2009. As a consequence of data gaps, we combined quarterly estimated consumption from 2009 and 2010 (Table 1) to estimate a yearly average. Data gaps resulted in a slight seasonal bias toward dry season orders (Q2 and Q4) – 10, compared with 9 quarters of rainy season (Q1 and Q3) – however, the impact would be minimized as dry season orders immediately follow a rainy season.

The second challenge was miscalculation by the health worker of ‘estimated consumption' due to arithmetic errors. These errors in estimated consumption arise from a number of miscalculations including the addition of extra zeros, adding the closing balance instead of subtracting it or counting medicines that had not been recorded as received (this was especially the case for ACT). Where arithmetic errors were obvious, they were corrected.

Combining the estimated consumption together with information from the HIS, we were able to estimate the amount of unaccounted medicines and then to compare it with the values from 1999 for the same three classes of medicines (Table 2).

*Antimalarial*: The amount of unaccounted antimalarials was reduced from 60% in 1999 to 18%, 10 years later. The results are statistically significant at the 95% level.

*ORS*: The accountability of ORS appears to have deteriorated over the past 10 years from 64% unaccounted ORS in 1999 to 81% unaccounted ORS in 2009. The results are statistically significant at the 95% level.

*Anthelmintic*: The amount of unaccounted anthelmintic was reduced from 82% in 1999 to 71%, 10 years later. This too is statistically significant at the 95% level.

## Discussion

As medicines budgets typically constitute a large proportion of discretionary health spending, countries must ensure that the appropriate quantities of medicines arrive at health facilities on time, and once there should be dispensed according to medicines distribution and treatment policies. The current ILS was introduced to provide *routine reporting of data coupled with routine ordering of resupplies, which enhances accountability and provides the central level with data for decision making* (Amenyah *et al*, 2005), and indeed has the potential to do so. However, from the evidence presented here, not one of the surveyed health facilities studied routinely reported as part of the ILS and there was no uniform improvement in accountability for the three selected ‘tracer' medicines. Although accountability of anthelmintic and antimalarial medicines appears to have improved, the fact remains that still 71 and 18% are unaccounted, respectively, while the accountability for ORS appears to have deteriorated. Although reaching a level of 0% of unaccounted medicines would be ideal, counting and arithmetic errors are realistically likely to occur in administrative data. Therefore, for the purpose of this study, we set a level of 85% accounted medicines as acceptable. This level reflects the general uncertainty, inaccuracy and incompleteness of information available from routine reporting systems; it is a generous margin of error and not intended as a gold standard. The ILS did not reach this level and is therefore in need of a management response.

Considering that the MSD price of a tin of 100 tablets of albendazole is currently Tanzanian Shilling (TZS) 1600 (U.S.$ 1.00), TZS 11,000 ($ 7.09) for 100 ORS sachets (MSD price catalogue 2011) and the government recommended retail price of a subsidized dose of ACT is TZS 1000 ($0.60)(Yadav *et al*, 2012), then the yearly value of the unaccounted medicines in 2009 was $ 3630 for the six health facilities. Projected to the Rufiji District level, the annual value of unaccounted medicines would be around $ 31,500.

Because of the use of secondary data, which in some instances were incomplete, our study is subject to bias. For example, the six health facilities were not randomly selected but selected on the basis of accessibility to facilitate data collection. The implication of this could be that the health facilities were more likely to have a larger flow of medicines but also more frequented by patients; therefore, the bias could move in both directions. We also assume that each of the three ‘tracer' medicines is used for a single disease, yet this may not always be the case.

A possible limitation of our study design is that our methods rely on different data sources for the amount of consumed medicines; in 1999, this came directly from medicines dispensed, while in 2009 this was derived from estimated consumption. Although demand-driven (number of patients) information was collected the same way in both periods (using summary HIS records), we were only able to verify the accuracy of the 1999 data. The health information data may have deteriorated over the years as health workers become increasingly burdened with the rise in the number of vertical programs and may not see the purpose of accurately reporting data. Therefore, a likely contribution to the unaccountability of ‘tracer' medicines may be that not all patients seen were recorded.

Poor record keeping and late submission of ordering forms by health facility workers has been found by others (Euro Health Group, 2007a; Chimnani *et al*, 2010; GIZ & Tanzanian German Programme to Support Health TGPSH, 2011). The lack of capacity of health facility staff to correctly order and manage medicines was also reported in a GIZ-funded project assessing 87 health facilities in four regions in 2011 (GIZ & Tanzanian German Programme to Support Health TGPSH, 2011). In addition, the GIZ report found that in some cases during redistribution of medicines by the District Pharmacists medicines ledger books were not adjusted. The risk of mistakes in ordering at the health facility level is accentuated in the case of ACT due to the four separate doses (based on patient weight) and because of the seasonality of malaria. Ordering mistakes could have contributed in part to the frequent periods of ACT stock-out, which Tanzania has experienced since 2009 together with other factors such as delayed procurement and distribution by the MSD due to lack of funds or capacity.

On the demand side, another limitation could be the changes in clinical treatment guidelines where, for example, in the past albendazole was more readily dispensed, or changes in patient demands may have resulted in patients receiving a dose of ACT, ORS or albenzaole even if they came into the health facility for another purpose.

A contributing factor to some of the ‘tracer' medicines not being fully accounted for could be leakage along the medicines supply chain, perhaps, involving direct pilferage. Incidences of theft of medicines at the health facility level in Tanzania have frequently been reported in the press (The Citizen, 2011; Siyame, 2012a); for example, the *Daily News* recently described the arrest of several pharmacists from a pharmacy owned by employees of the regional hospital who were discovered selling medicines intended for the public sector, especially malarial medicines (Siyame, 2012b). The Audit Report on Global Fund grants to Tanzania in 2009 reported a comparison between number of malaria cases and estimated ACT consumed, which found nearly twice the amount of ACT consumed for the number of malaria patients, suggesting a ‘leakage' in the ACT medicines delivery (The Global Fund to Fight AIDS Tuberculosis and Malaria, 2009). ACTs also have a much higher resale value than previous antimalarials (chloroquine) and the street value of ACT would have increased during periods of national public sector stock-outs, which may increase the incentive for pilferage. Such leakages have also been found in other countries; a study by McPake *et al* (1999) in Uganda using similar methods to ours together with qualitative evidence found very high medicines leakages, which resulted in weaker health worker performance.

Our results are inconclusive whether the ILS is better or worse, but emphasize the point that both systems clearly reveal an unacceptable accountability gap. Two general obstacles could explain this, the first is complexity in the design of the logistics system and the second being its governance. Design weaknesses in the ILS include an increased work burden on staff by requiring them to make many difficult calculations for over 100 products every 3 months and submit forms in person to the district capital. Going to the district capital could mean over a day's travel for those working in some remote locations, which would leave these health facilities without staff during the travel period. Limitations of health worker capacity were also found during an evaluation of the ILS pilot in Dodoma and Iringa regions in 2005 where health workers were failing to fill in requests for all priority medicines and to submit the R&R forms on time (Amenyah *et al*, 2005). These problems could be mitigated through increased training of staff and if the ordering was done using mobile phones or digital devices and the ordering system was simplified, for example, with calculations of estimated consumption being done only once a year and quarterly deliveries from the MSD being based on these estimates.

The ILS was designed to integrate the vertical programs with the essential medicines program, but certain vertical programs such as TB, AIDs and vaccines still operate separately. These items are delivered through their respective vertical programs, with additional reporting systems, which have the potential to create further confusion and workload for health workers. Integrating these programs into the ILS would avoid parallel systems and reduce the burden on health workers to report separately. Design failures of supply chains in low-income countries have been identified as one of the most important barriers to access to medicines (Kraiselburd & Yadav, 2012).

Regarding governance, the ILS appears to have limited accountability structures. For instance, no individual (health facility worker or district health official) is held accountable if an ILS form is not submitted or if repeated mistakes in calculations are being submitted to the MSD; gaps in the data hamper efforts toward improving accountability. If no order is placed, then no medicines arrive with the ultimate consequence that the community goes without medicines. Lack of district oversight was also found in the 2010 evaluation by Chimnani *et al* (2010). Our study also found cases where the district resubmitted old forms with new dates. Achieving accountability requires a degree of transparency, although the ILS is designed to increase transparency, if health workers do not complete forms adequately, then the ILS cannot provide information on medicines distribution once at the facility level. Data reconciliation with the HIS (MTUHA) as done in this study would be a simple way to check the plausibility of medicines ordered. Introducing systematic data reconciliation between the ILS and the MTUHA would greatly improve information on rational medicines consumption. Without the ability of the ILS to fully account for medicines ordered, delivered, prescribed and used, the system will continue to suffer from inherent inconsistencies combined with increasing vulnerability and negligence. In the case of essential life-saving medicines such as ACT, the need for accountability is increased to ensure avoidable mortality is reduced.

At present, Tanzania is investing significant resources toward a mHealth strategy, which will strengthen some of the limitations of the ILS under the ‘ILS Gateway' model, a USAID-funded project. The ILS Gateway is a mobile phone-based alert and reporting system for the supply and logistics of 20 essential health commodities and is being piloted across 1600 public health facilities. The mHealth rollout will also include other disease-monitoring initiatives using mobile phones. Together, these initiatives should make reconciliation of data easier and highlight inconsistencies. Another recent change is that the MSD will deliver directly to health facilities (bypassing the DMO), making the MSD fully accountable for the entire supply chain. This, in combination with the mHealth strategy, is encouraging considering the increasing number of new initiatives (medicines donations and low-cost access initiatives) together with an expected rise in the number of health facilities (7000 by mid 2013), both of which may augment the workload and complexity at the MSD, increasing the need for a reliable reporting system.

## Conclusion

The availability of medicines at health facilities is a critical element of service delivery quality, without which the districts will be seriously limited in their ability to provide adequate health care. To our knowledge, this study is the first to critically examine the availability of medicines under the current logistics supply system compared with the previous kit system. This study suggests that there is an opportunity to reconcile information on the demand for essential medicines with their supply. This approach could be a way of exploring the accountability of resources in a health system, which was not exploited under either medicines delivery system. Of the three medicines we compared, absolute accountability rates were still low in the ILS, with around 20 to 80% of medicines not being accounted for, and with one tracer (ORS) experiencing a deterioration in accountability compared with the previous kit system. Such degrees of unaccountability in the distribution of medicines suggest that the ILS is unable to effectively monitor the supply and use of medicines, thus facilitating a health system environment in which obfuscation can occur and in which performance can go unrewarded. Although the ILS was designed to increase accountability and to reduce wastage of resources, its overly complex and ‘paper-driven' design together with other factors such as limited regular staff training and supervision has constrained it from fully achieving these targets. As essential medicines constitute a key component of service delivery quality, which in turn is critical for improving effective access, urgent system design and governance interventions need to be developed to fundamentally strengthen this critical aspect of the health system.

## Authors' contributions

DDS and IML contributed equally to the conceptualization and design of the approach. PC, GR, DDS, HK and CM led the work in the field in 1999 and IML and DDS led the fieldwork in 2009. DDS and IML managed and analyzed data. IML, DDS, PC and GR wrote the manuscript and all authors reviewed, contributed to and approved the final manuscript.

## Figures and Tables

**Table 1:**
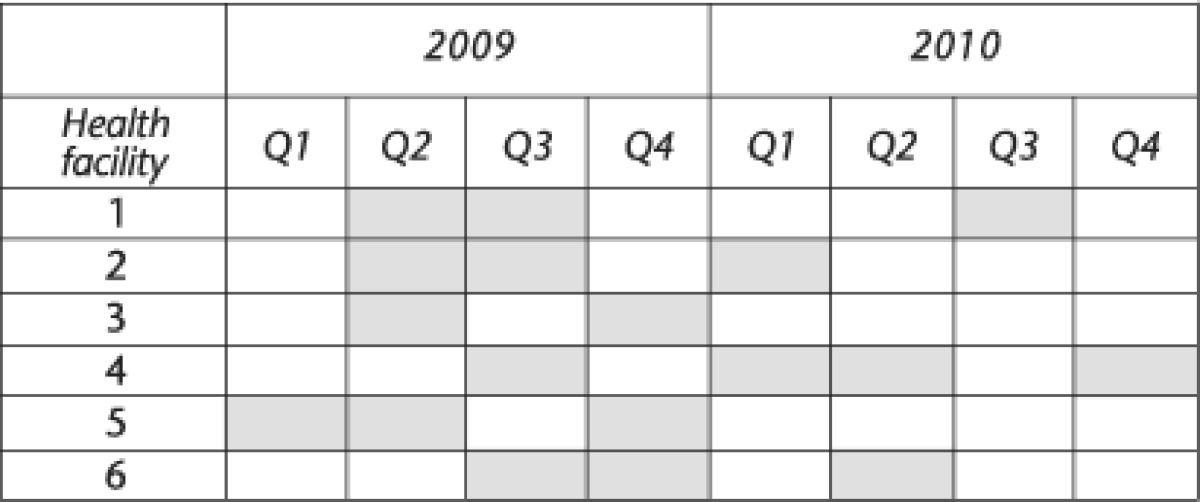
Quarterly R&R forms submitted across six health facilities, Rufiji, 2009 and 2010

**Table 2:** Percentage of unaccounted medicines (anthelmintic, antimalarial, ORS) in 1999 and 2009 across six public health facilities in the Rufiji District, Tanzania

*Medicine*	*1999 (95% CI)*	*2009 (95% CI)*
Antimalarial	59.8% (59.7–60.0)	17.8% (17.5–18.2)
ORS	63.8% (63.1–64.5)	80.7% (80.1–81.3)
Anthelmintic	81.9% (81.6–82.1)	71.1% (70.2–72.0)
